# Cross Modal Few-Shot Contextual Transfer for Heterogenous Image Classification

**DOI:** 10.3389/fnbot.2021.654519

**Published:** 2021-05-24

**Authors:** Zhikui Chen, Xu Zhang, Wei Huang, Jing Gao, Suhua Zhang

**Affiliations:** ^1^The School of Software Technology, Dalian University of Technology, Dalian, China; ^2^The Key Laboratory for Ubiquitous Network and Service Software of Liaoning Province, Dalian, China; ^3^Department of Critical Care Medicine, First Affiliated Hospital of Dalian Medical University, Dalian, China

**Keywords:** few-shot learning, deep transfer learning, context awareness, cross modal information, image classification

## Abstract

Deep transfer learning aims at dealing with challenges in new tasks with insufficient samples. However, when it comes to few-shot learning scenarios, due to the low diversity of several known training samples, they are prone to be dominated by specificity, thus leading to one-sidedness local features instead of the reliable global feature of the actual categories they belong to. To alleviate the difficulty, we propose a cross-modal few-shot contextual transfer method that leverages the contextual information as a supplement and learns context awareness transfer in few-shot image classification scenes, which fully utilizes the information in heterogeneous data. The similarity measure in the image classification task is reformulated via fusing textual semantic modal information and visual semantic modal information extracted from images. This performs as a supplement and helps to inhibit the sample specificity. Besides, to better extract local visual features and reorganize the recognition pattern, the deep transfer scheme is also used for reusing a powerful extractor from the pre-trained model. Simulation experiments show that the introduction of cross-modal and intra-modal contextual information can effectively suppress the deviation of defining category features with few samples and improve the accuracy of few-shot image classification tasks.

## 1. Introduction

In the age of big data, data are vast but sometimes precious. Deep neutral networks have achieved great performance when sufficient samples are offered. However, when it comes to practical tasks, these frameworks fail due to small data limitations. Insufficient data appear due to difficulty in acquisition, confidentiality, privacy, the high cost of labeling, or other reasons. Few-shot learning is proposed to simulate a human learner who is adept at generalizing categories with a handful of samples, and it fits well with a possible lack of data in the task of actual solutions (Lu et al., 2020), leading to an innovation of artificial intelligence algorithms.

However, a major challenge in few-shot learning is that unseen categories or target categories trained with few samples might be affected by the specificity of a particular sample, which misleads a false bias toward local features and a lack of diversity of intra-class features. Since deep transfer learning (DTL) has been a good solution in actual tasks to transfer and reuse knowledge from the auxiliary domain, researchers have been exploring whether some important information can be transferred in few-shot learning in a similar way to remedy the problem. Chen et al. (2020a) proposed diversity transfer, which transfers latent diversity from known categories and supports features to generate novel samples with diversity. Chen et al. (2020b) found that the information of correlated categories is able to be transferred in few-shot recognition, which can significantly help learn new categories while avoiding being too influenced by specificity, and incorporating semantic correlations between categories can regularize this kind of transfer. Zhou and Mu (2020) used meta-learning to transfer large-scale richly annotated image data between domains for few-shot video classification. Xu and Du (2020) learned transferable features in the task of few-shot text classification. In general, although there is no consensus on “what to transfer” in few-shot learning, the introduction of DTL has effectively improved performance and narrowed the gap with human learners. Meanwhile, the schemes can often transfer important information across the modal, which provides a good prospect for processing heterogeneous data, since the data are often heterogeneous in the application scenario.

Context-aware techniques can further inhibit specificity and remove mismatches with increasing accuracy thanks to the use of important contextual information. Yang et al. (2020b) took the lead in proposing Context-Transformer, which exploits context from few-shot training samples under a deep transfer framework. It discovers clues from the background of the images in the target domain to reduce object confusion in the object detection task, enhancing the discriminative power of the detector. Inspired by the research, this idea can be easily extended to tasks such as image classification. However, there is not always important information we need in the background. On the contrary, sometimes the background is a solid color or made of fixed shapes, and it is even possible to generate negative feedback noise.

**Figure 1:**
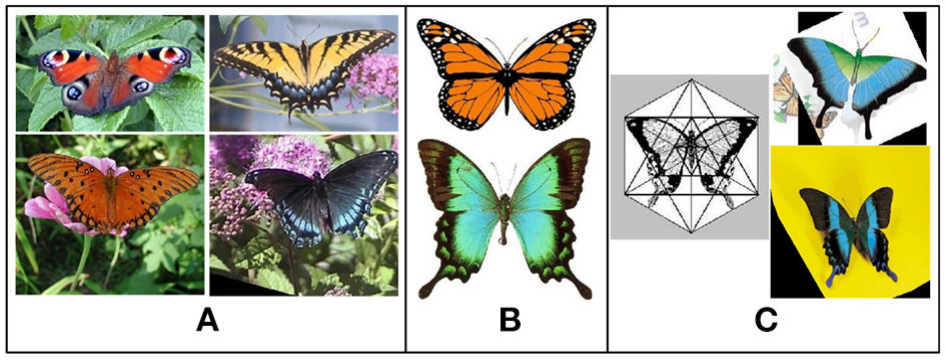
Image samples of class “butterfly” in Caltech-101. **(A)** Images with contextual information in the backgrounds. **(B)** Images with blank backgrounds. **(C)** Images with negative-feedback information in the backgrounds.

For example, as illustrated in Figure 1, the images are all from the class “butterfly” in the dataset of Caltech-101 (Li et al., 2006). In the A section, the flowers and leaves in the background can contribute contextual information to better recognize the butterflies in the picture. However, in section B, no more information can be provided and the background in C might have a negative impact. Such composition scenarios in classes of image datasets should be common. Furthermore, what if a butterfly perches on a newspaper or album with pictures of cars, buildings, or streets?

Thus, sometimes the background information should be suppressed. And it makes sense that the inherent semantic information from textual features in heterogeneous samples should be more reliable, followed by the extracted information. Therefore, compared with seeking for clues in the pictures themselves, cross-modal contextual information is more taken into account in this paper, and the idea is extended into the image classification task, so that heterogeneous data samples can also be fully utilized. Furthermore, the extracted visual and semantic features can be embedded into the similarity measurement process in a more reasonable way by a sequence model such as LSTM. And the DTL scheme can be also introduced for providing a more powerful extractor by reusing it in pre-trained vision models that have rich experience in recognizing local visual patterns.

The main contributions of this paper are summarized as follows:

- A more efficient metric is designed so that the Pearson correlation coefficient is used in measuring the similarity of image classification under the scenario of few-shot learning.
- The cross-modal contextual information is taken into account to recognize representative features, inhibiting sample specificity under few-shot scenes. A joint similarity metric is introduced for integrating the visual similarity and semantic similarity in heterogeneous samples together.
- The DTL scheme is used to optimize the extracting process in the model for better recognizing local visual features.
- Simulation experiments and ablation experiments are designed to verify the effectiveness of the proposed method in the image classification task of few-shot learning.

## 2. Related Works

### 2.1. Few-Shot Learning

Few-shot learning, which simulates a human's capability to learn and generalize from very few data samples, is considered one of the solutions to achieve advanced artificial intelligence (Wang et al., 2020b), in which the prior information can correspond to the known information or historical information in human learning. Existing few-shot learning strategies can be divided into metric learning-based methods (Snell et al., 2017; Qiao et al., 2018) to learn good metrics of the new task, meta learning-based methods (Munkhdalai and Yu, 2017; Franceschi et al., 2018) that train efficient meta-learners for fast adaptation, and augmentation-based (Gao et al., 2018) as well as generation-based (Schwartz et al., 2018; Zhang et al., 2018) methods that try to alleviate the lack of training samples directly. Inspired by them, various methods are designed for the image classification task, because researches on computer vision have always been a touchstone thanks to the intuitiveness and intelligibility of data (Ren et al., 2018; Lifchitz et al., 2019).

The proposed method can be categorized into an augmentation-based few-shot learning strategy, in which the augmentation mainly occurs at a deeper level to upgrade the generalization ability. Nowadays, supervised feature-level augmentation for training samples in a task has been achieved, such as Attribute-Guided Augmentation (AGA) (Dixit et al., 2017), Dual TriNet (Chen et al., 2019), and the Attribute-Based Synthetic Network (ABS-Net) (Lu et al., 2018). Augmentation always depends on external information, such as term vectors and semantic attributes. In this paper, the cross-modal context information is used as an innovative scheme.

### 2.2. Deep Transfer Learning

In augmentation-based few-shot learning, augmentation is an intuitive way to alleviate the lack of training samples and data diversity. Meanwhile, the DTL is also known as a common strategy for insufficient data samples in training deep models. It is natural to consider a combination of them, however, which specific parts can be transferred in few-shot learning is not settled yet. The Feature Trajectory Transfer (FTT) (Kwitt et al., 2016) transfers the consecutive attributes in images into the synthetic features in a one-sample class, and then the Feature Space Transfer Network (FATTEN) (Liu et al., 2018) provides an end-to-end optimization scheme of it. D2N4 (Yang et al., 2020a) uses transfer learning in the few-shot space target recognition for a meta-learning paradigm that quickly adapts to new tasks. The transfer can also be used in metric-based classification (Tseng et al., 2020) for augmenting the image features. Although the known samples are limited in few-shot learning, the DTL can efficiently enrich the prior information with reasonable reuse. However, comparing it to the traditional application scenario between visible categories with sufficient samples, the transfer into unseen categories or target categories in few-shot scenarios needs more consideration.

### 2.3. Context-Aware Technique

In few-shot learning, the lack of diversity possibly affects the unseen categories or target categories by limited sample specificity. Even if the samples belong to the same class, different samples of images might show very diverse features. Thus, the introduction of contextual information helps to provide supplementary knowledge for classification accuracy. The context-aware technique is widely used in natural language processing (NLP) tasks such as text classification (Xu et al., 2020), while few-shot learning is often used in image classification, and it has been a meaningful challenge in a modeling context in computer vision tasks (Bell et al., 2016; Kantorov et al., 2016). Research has been proposed to achieve object detection (Chen and Gupta, 2017; Wang et al., 2018) and image classification (Zhang et al., 2020) with contexts, and (Kamara et al., 2020) combines contextual information in time series classification. Regarding few-shot learning, the Context-Transformer (Yang et al., 2020b) firstly investigates the context in object detection. And it will be a long-term challenge to effectively reduce learning difficulty by exploiting the guidance of contextual knowledge.

## 3. Method

**Figure 2:**
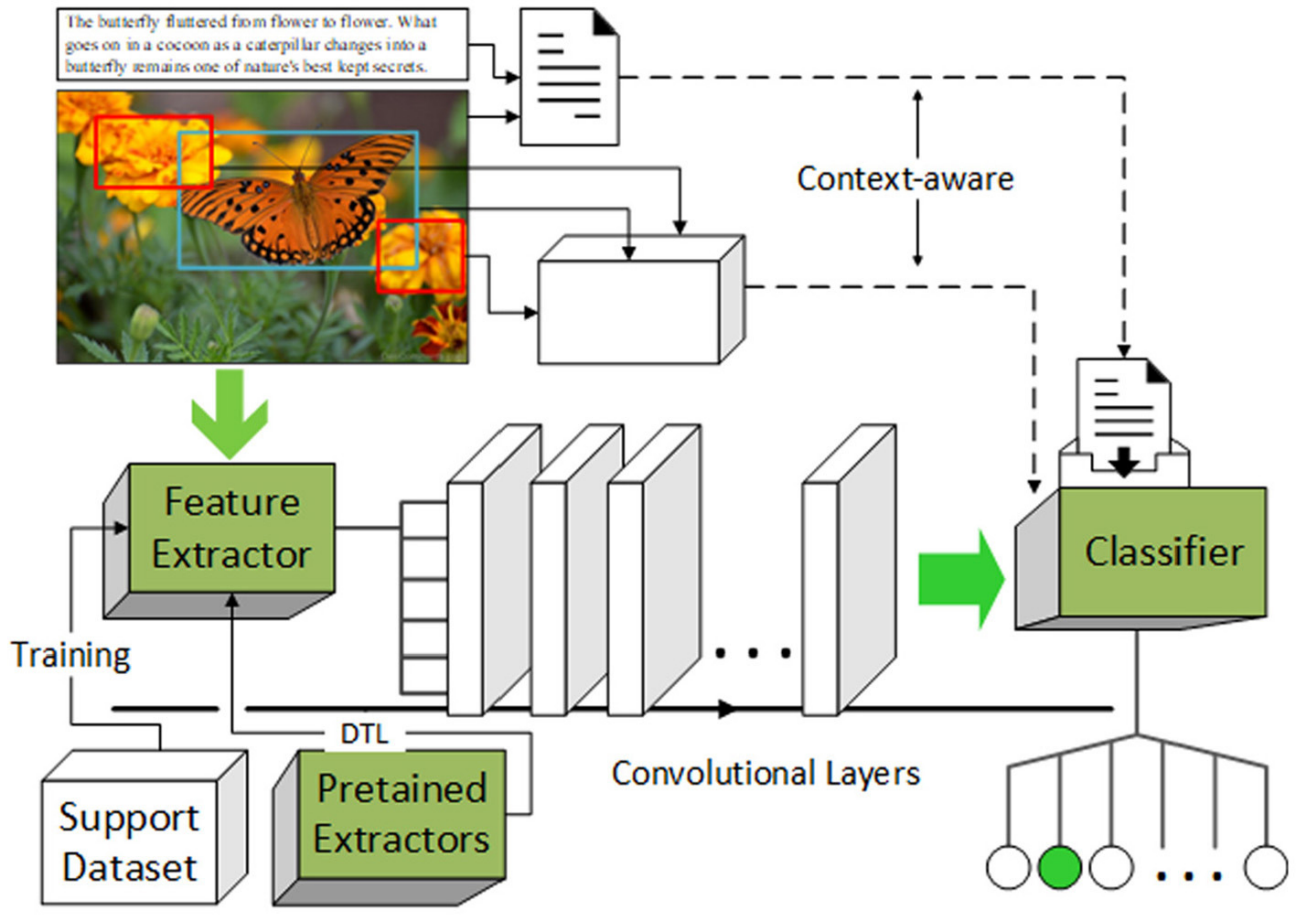
Architecture of proposed model.

Classical convolutional neural network (CNN)-based image classification tasks often fail in directly using a few-shot learning scenario, since the models rely on well-trained feature extractors and classifiers, which need a large number of samples to adequately learn interior features. The architecture of the proposed model is designed to adapt few-shot learning tasks: (1) For the feature extractors, the idea of DTL is introduced to help gain more information in feature learning, not only from samples in the support set. (2) For the classifiers, two kinds of context-aware techniques are combined to inhibit the bias toward the specificity of samples, gain information from the background while avoiding the negative feedback from the insignificant background. (3) On the basis of the improvements above, the structure of the network is optimal designed. The architecture of the proposed model is illustrated in Figure 2.

A typical CNN-based image classification model will co-optimize the feature extractor and classifier during the training process, and the images are divided into the correct categories in the end. To be specific, for each input picture sample (defined as *x*), its feature representation is learnt through the feature extractor and an output of 𝕏. Let *M* be the number of categories, then a function *p*_*M*_(𝕏) = *w*_*M*_𝕏 + *b*_*M*_ can be introduced to measure the possibility that *x* belongs to each category through the classifier, *w*_*M*_ and *b*_*M*_ are the weight and bias term. Thus, the model can label each *x* by calculating $\arg\underset{M}{\max}P_{𝕃\in M}\left(x\right)$ so that

(1) $$\begin{array}{l} P_{𝕃\in M}\left(x\right)=\frac{\exp\left(p_{M}\left(𝕏\right)\right)}{\sum_{n=1}^{M}\exp\left(p_{M}\left(𝕏\right)\right)} \end{array}$$

in which 𝕃 stands for the label of the picture. And formula (1) can be reformulated to relax its constraints so that many other metrics can be introduced to gain better performance, such as

(2) $$\begin{array}{l} \arg\underset{M}{\max}\left[\frac{\exp\left(p_{M}\left(𝕏\right)\right)}{\sum_{n=1}^{M}\exp\left(p_{M}\left(𝕏\right)\right)}\right]=\arg\underset{M}{\max}p_{M}\left(𝕏\right) \end{array}$$

and

(3) $$\begin{array}{l} \begin{array}{l} p_{M}\left(𝕏\right)=w_{M}𝕏+b_{M}\\ \;=\frac{1}{2}\left(∥w_{M}{∥}_{2}^{2}+∥𝕏{∥}_{2}^{2}-∥w_{M}-𝕏{∥}_{2}^{2}\right)+b_{M} \end{array} \end{array}$$

which seems like a typical similarity measure if *b*_*M*_ = 0. Then,

(4) $$\begin{array}{l} \begin{array}{l} \arg\underset{M}{\max}P_{𝕃\in M}\left(x\right)=\arg\underset{M}{\max}p_{M}\left(𝕏\right)\\ \;=\arg\min∥w_{M}-𝕏{∥}_{2}^{2} \end{array} \end{array}$$

whereupon the label prediction is converted into seeking the minimum distance between *w*_*M*_ and 𝕏, so that a specific metric can be introduced to measure the similarity better.

In the proposed method, we use the Pearson correlation coefficient to reformulate *p*_*M*_(𝕏). Because the original heterogeneous data will exhibit high-dimensional characteristics, especially when contextual features are taken into account, while the standardization and the zero-centered operation in Pearson's coefficient are more suitable for high-dimension and missing-dimension scenarios. We also designed comparative experiments to compare the performance between using the typical Cosine and Euclidean metrics, as shown in Figure 7. Thus, *p*_*M*_(𝕏) transforms into:

(5) $$\begin{array}{l} \begin{array}{l} p_{M}\left(𝕏\right)=\frac{w_{M}^{*}{𝕏}^{*}}{∥w_{M}^{*}{∥}_{2}∥{𝕏}^{*}{∥}_{2}} \end{array} \end{array}$$

in which ^*^ is the zero-centered operation so that

(6) $$\begin{array}{l} {𝕏}^{*}=𝕏-\frac{1}{x}\overset{x}{\underset{n=1}{\sum }}X_{n},X_{n}\in 𝕏 \end{array}$$

and is the same as $w_{M}^{*}$.

However, the conversion also makes it more susceptible to the specificity of the sample, especially in the scenario of few-shot learning. In this paper, we use context-aware techniques to help the classifier inhibit the negative effects of specificity. Meanwhile, the introduction of the DTL scheme in helping feature learning using extractors can inhibit the effects of specificity to some extent, because it can reuse the information of many universal features which have been well-learned.

### 3.1. Context-Aware Techniques in Classifier

In the scenario of the image classification task, in order to include the background information taken by object detection in the model, we use Faster R-CNN (Ren et al., 2017) that identifies a set of objects 𝕏_*O*_ from input *x*, in which each *x*_*On*_ ∈ 𝕏_*O*_ consists of the visual feature vector *v*_*n*_ and spatial feature vector *s*_*n*_ = (*x, y, w, h*) with its label *l*_*On*_. However, we do not really need the spatial information of where these objects are located so *s*_*n*_ is discarded and ${𝕏}_{O}=\left\{v_{O}\in {\text{R}}^{d},l_{O}\right\}$ so *d* represents the dimensions of the visual feature vector. The 𝕏_*O*_ in input *x* will serve as the auxiliary information that measures the similarity between *x* and the samples in the corresponding correct target category with a certain weight.

**Figure 3:**
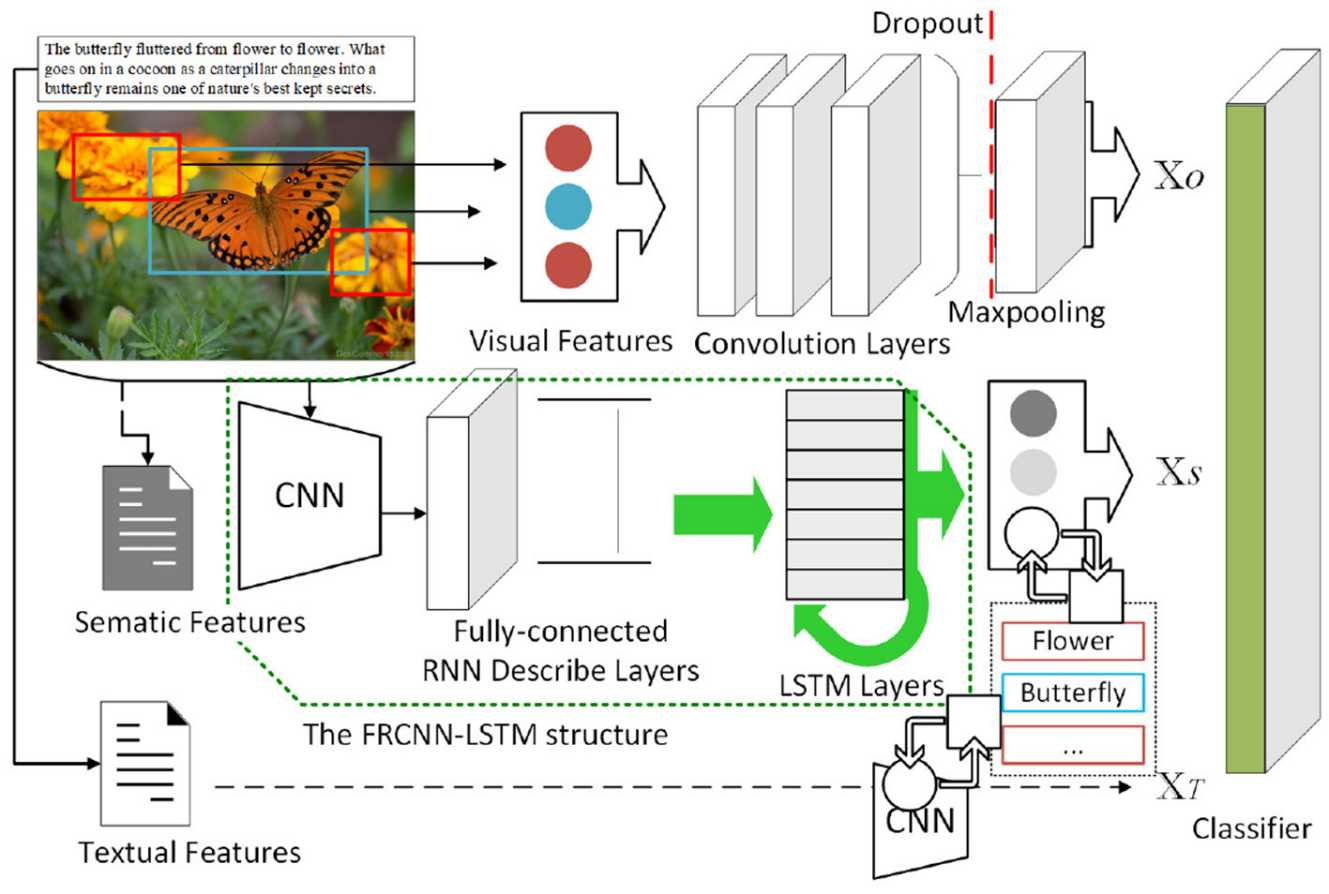
Architecture for jointly using contextual information.

In addition, semantic information can also be integrated as an important supplement to avoid the negative feedback caused by solid color, meaningless background, and misleading noise in classification. In the proposed method, the semantic information can be divided into implicit and explicit information: implicit semantic information is a feature vector set of semantics 𝕏_*S*_ contained in the images, which can be extracted through an FRCNN-LSTM structure. And explicit semantic information is used to consider whether the heterogeneous support datasets themselves contain textual information and utilize their semantic features, a combination of the LSTM and a CNN model can be used as an effective scheme to realize semantic embedding, converting the label to the corresponding 𝕏_*T*_ in reverse. Thus, the cross-modal contextual information is introduced to make better use of information from heterogeneous data, which helps jointly measure the similarity between few-shot samples and the target domain, preventing over-bias caused by sample specificity. The schematic diagram is shown in Figure 3.

The high-level abstraction of visual objects in images and their associated natural language can provide the necessary semantic information. Johnson et al. (2016) proposed DenseCap, which generates dense and rich annotations of the input image, we use their idea to build an FRCNN-LSTM structure for extracting 𝕏_*S*_: Each input image will first be processed through a CNN for region sampling, and then the Faster R-CNN ensures the same size feature representation will be extracted for each region proposal, so that the information can be processed with a fully connected recognition network and described with an RNN language model. The LSTM recurrence is used for realizing the RNN language model. However, the visual features will be intercepted before generating annotations, and extracted as one of the similarity metric together with 𝕏_*O*_ in the comparison.

The combination of the LSTM and a CNN model has been proved to be an effective scheme to realize semantic embedding and similarity measurement aided by sequence information (Koller et al., 2020; Wang et al., 2020a), and we use it to find the local visual feature presentation 𝕏_*T*_ of the corresponding explicit textual information. The embedded LSTM layer will first accept the semantic information of text embedding as input, and the output of the LSTM layer is then input into convolution layers which are expected to extract 𝕏_*T*_. The LSTM layer acts like an editor who provides semantic information as tags helping the CNN layers to use a richer representation of the original input to find local visual patterns with the corresponding textual features.

To sum up, we can measure the similarity of each input image from three dimensions in the CNN layers by comparing 𝕏_*O*_, 𝕏_*S*_, and 𝕏_*T*_, since the prediction of labels depends on extracted features, and the semantic similarity is positively correlated with the feature similarity between samples. Furthermore, the contribution weight of the above contextual information is adjusted. We believe that the inherent semantic information 𝕏_*T*_ from textual features in heterogeneous samples is the most reliable, followed by the extracted semantic information 𝕏_*S*_ and object detection information 𝕏_*O*_. Thus, a joint similarity metric is introduced that integrates the feature similarity between data with their semantic similarity:

(7) $$\begin{array}{l} 𝕊=\{\begin{array}{ll} \left(\alpha {𝕊}_{S}+\beta {𝕊}_{O}\right)\left(1+\left({𝕊}_{T}-\left(\alpha {𝕊}_{S}+\beta {𝕊}_{O}\right)\right)\right) & \left({𝕊}_{T}\neq 0\right)\\ \alpha {𝕊}_{S}+\beta {𝕊}_{O} & \left({𝕊}_{T}=0\right) \end{array} \end{array}$$

in which 𝕊_*O*_/𝕊_*S*_/𝕊_*T*_ can be equally regarded as the modal similarity of the image measured from three specific dimensions. By loosening their constraints as described above, they are calculated by formula (5), respectively, so that

(8) $$\begin{array}{r} {𝕊}_{O}=p_{M}\left({𝕏}_{O}\right)=\frac{w_{M}^{*}{𝕏}_{O}^{*}}{∥w_{M}^{*}{∥}_{2}∥{𝕏}_{O}^{*}{∥}_{2}}\;,\\ \text{and}{𝕊}_{𝕊}\text{, }{𝕊}_{T}\text{ in a similar way.} \end{array}$$

And parameters α and β are used to adjust the weight of extracted contextual information. Equation (7) is designed to maximize the original semantic information so that a larger 𝕊_*T*_ can lead to a larger 𝕊, unless there is no textual information in the original samples (𝕊_*T*_ = 0). In this way, the semantic feature information can be integrated together and the use of cross-modal textual information in raw heterogeneous data is maximized.

Finally, the output of the convolutional layers will be gathered and the final output will be the classification labels. And the function in the original CNN-based image classification task translates to:

(9) $$\begin{array}{l} \arg\underset{M}{\max}\left(p_{M}\left(𝕏\right)+𝕊\right). \end{array}$$

To sum up, the introduction of contextual information alleviates the influence by sample specificity in few-shot learning.

### 3.2. Deep Transfer Scheme in Extractor

In addition to transferring contextual information, although few-shot learning limits the number of training samples, the DTL scheme can circumvent the limit by reusing a powerful pre-trained feature extractor with its recognition pattern in the corresponding convolutional layers, which had remembered the ability to recognize local patterns from homogeneous instances. The scheme of few-shot learning can take advantage of the ideology in transductive learning so that features from unclassified samples in unseen categories or a target category which are used in training are able to help the extractor extract the representative features of the corresponding category.

Specifically, (1) convolutional layers acting as an extractor in CNN architecture can be reused so that the layers in front have learnt to extract universal visual features which are applicable for all kinds of images, and the latter layers can express highly abstract data-specific features. Parameters in the front layers are frozen and in the latter layers will be fine-tuned to fit samples in a specific task on the basis of the DTL scheme, to cope with the under-fit and low performance in the event of not enough training data. (2) Similarly, convolutional layers in RCNN-LSTM architectures act as the auxiliary domain to extract background information from images can be reused by DTL since the learning ability of the deep neural network is not able to contribute in the event of insufficient training data. And in this way, it reduces the cost needed for extra training on an object detection branch to achieve feature vector extraction in the model.

## 4. Simulation Experiments

### 4.1. Dataset Settings

In this section, we evaluate the proposed methods used by the miniImageNet, CUB, and Caltech-101 datasets.

The miniImageNet dataset (Vinyals et al., 2016) has been widely used by researchers to evaluate the performance of computer vision methods under few-shot scenarios, and we use the settings similar to the schemes in state-of-the-art methods so that the original dataset is divided into 64 training categories, 20 testing categories, and 16 validation categories, optimizing hyper parameters in the validation set to help the training set obtain better final results.

The CUB (Wah et al., 2011) dataset, often used to evaluate the performance of few-shot learning algorithms, contains 200 categories of birds and is divided into training, validation, and testing sets with 100, 50, and 50 categories following the common settings in Schwartz et al. (2018).

The Caltech-101 dataset is commonly used in training and testing traditional computer vision recognition and classification models, consisting of 9,146 images that are divided into 101 different categories and an additional clutter category. We designed a validation scheme to use the Caltech-101 dataset in few-shot image classification task evaluation, because: (1) in Caltech-101, most of the chosen images have varying degrees of background clutter, which may potentially lead to incorrectness in traditional algorithm training. Our method aims at using contextual information wisely while suppressing useless and negative-feedback information in the background, so the background clutter in samples of Caltech-101 can help to compare the effectiveness of the proposed scheme. (2) Some categories in Caltech-101 only contain a small number of image samples (even less than 31), which is inadequate for training for a traditional task, but can be seen as a typical few-shot learning scenario. It is divided into training, validation, and testing sets with 50, 26, and 25 categories.

### 4.2. Comparative Results

Firstly, the comparative results with the previous state-of-the-art methods on the miniImageNet dataset are shown in Table 1 to evaluate the performance of the designed model. Since the miniImageNet dataset lacks the corresponding description text, it can only verify when using the implicit semantic information in the image for context transfer. All results are reported with 95% confidence intervals, using a standard *N Way K Shot* classification task under a few-shot scenario as defined in Vinyals et al. (2016). Concerning the architecture of the training model with the proposed contextual transfer scheme, we choose the same network structures as each previous method we used (when possible) for comparison, because the convolutional layers for feature learning can be flexibly adjusted in the proposed architecture, such as the designed CNN net with four convolutional modules (in Vinyals et al., 2016; Finn et al., 2017; Ravi and Larochelle, 2017; Chen et al., 2020a) and the ResNet-12 (the rest), while the DTL scheme is introduced for adaptation. The experiment results show that the proposed method can perform competitive results that outperform the previous method under both the 5 *Way* 1 *Shot* and 5 *Way* 5 *Shot* setting, which preliminarily verifies the feasibility and effectiveness of the proposed scheme.

**Table 1:** Performance comparisons of few-shot image classification on miniImageNet.

**Method**	**In**	**Acc**	
		**5 Way 1 Shot**	**5 Way 5 Shot**
Matching network (Vinyals et al., 2016)	NIPS (2017)	43.56 ± 0.84	55.31 ± 0.73
Meta-Learning LSTM (Ravi and Larochelle, 2017)	ICLR (2017)	43.44 ± 0.77	60.60 ± 0.71
Model-agnostic Meta learning (Finn et al., 2017)	ICML (2017)	48.7 ± 1.84	63.11 ± 0.92
Delta-encoder (Schwartz et al., 2018)	NIPS (2018)	59.9 ± 0	69.7 ± 0
Rapid adaptation Resnet (Munkhdalai et al., 2018)	ICML (2018)	56.88 ± 0.62	71.94 ± 0.57
DTN (Chen et al., 2020a)	AAAI (2020)	57.89 ± 0.84	73.28 ± 0.65
STA Net (Yan et al., 2019)	AAAI (2019)	58.35 ± 0.57	71.07 ± 0.39
TPN (Liu et al., 2019)	ICLR (2019)	59.46 ± 0	75.65 ± 0
LEO (Rusu et al., 2019)	ICLR (2019)	61.76 ± 0.08	77.59 ± 0.12
Contextual transfer (Proposed method)	/	62.27 ± 0.76	77.81 ± 0.98

Furthermore, as Table 2 shows, simulation experiments are designed on the datasets CUB and Caltech-101 to verify that the proposed method can be used in various few-shot learning scenarios. In particular, for these two datasets, we consider the textual modal's contextual transfer. Since the original samples in the dataset are not heterogeneous ones, textual information are manually added to simulate and verify the ability of the proposed method in processing heterogeneous data and utilizing cross-modal contextual information. As for the CUB dataset, we take advantage of its intrinsic set of attributes as labels. And DenseCap (Johnson et al., 2016) is used to help generate a series of annotations for Caltech-101.

**Table 2:** Performance comparisons of few-shot image classification on CUB, Caltech-101, and textual modal added.

**Method**	**CUB**		**Caltech-101**	
	**1 Shot**	**5 Shot**	**1 Shot**	**5 Shot**
Matching network (Vinyals et al., 2016)	49.3	59.3	37.6	51.3
Meta-learning LSTM (Ravi and Larochelle, 2017)	40.4	49.7	43.2	57.2
Model-agnostic meta learning (Finn et al., 2017)	38.4	59.1	35.6	52.3
Delta-encoder (Schwartz et al., 2018)	69.8	82.6	66.0	80.7
Deep DTN (Chen et al., 2020a)	72.0	85.1	69.6	83.3
Contextual transfer (Proposed method)	72.2	85.7	70.1	84.2
Contextual transfer (Textual added)	75.1	87.8	76.1	86.3

**Figure 4:**
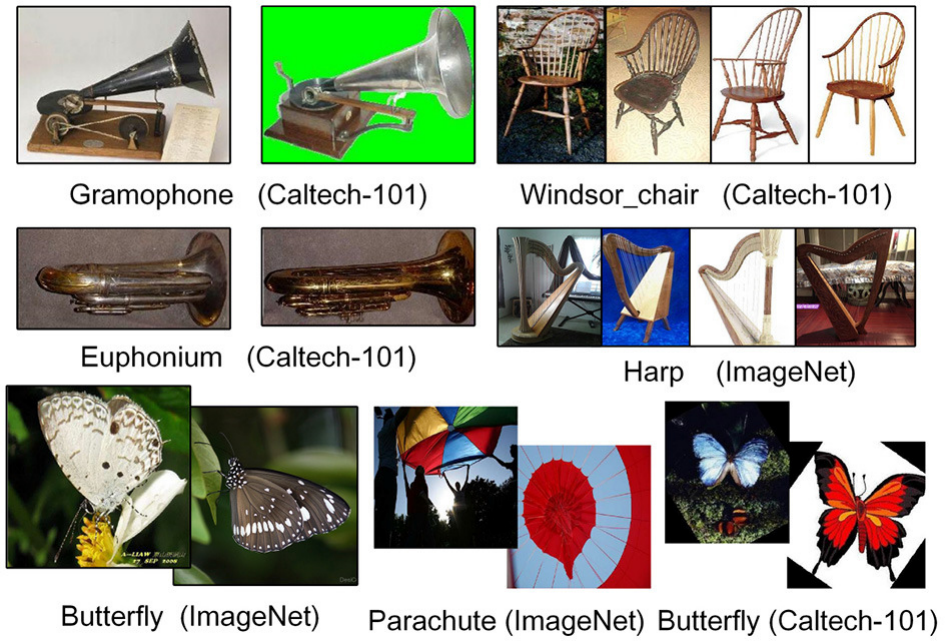
Sample similarity in few-shot scenarios.

Moreover, in the analysis of experimental results, we believe that contextual information plays an extraordinarily important role in some scenarios. Consistent with the previous discussion, when no contextual information is used, the image classification task under a few samples scenario tends to be misclassified into categories that are similar to the morphological features of particular samples. For example, the samples in the category “gramophone” and the “euphonium” of the Caltech-101 dataset are sometimes confused and put into the wrong category, since they are similar from a certain angle, especially when images of different sizes are formatted uniformly for input into the model. Thus, the inference is that such similarities are more likely to occur between categories of different datasets, such as the “Windsor_chair” in Caltech-101 with the “harp” in ImageNet, and the “butterfly” with “parachute” (while the “butterfly” in ImageNet can be distinguished from “parachute”). Figure 4 shows an illustration.

Since only a small number of samples are used in the training of few-shot learning tasks, if such similarity is randomly selected, the results would easily be influenced by the specificity. Although we may not encounter this problem in a simulation experiment when the commonly used dataset is selected separately for validation. However, there are many categories with the same name in different datasets, and we believe that putting them together can better simulate the sample distribution in the real world, which preserves this kind of accidental similarity to validate the importance of contextual information.

**Figure 5:**
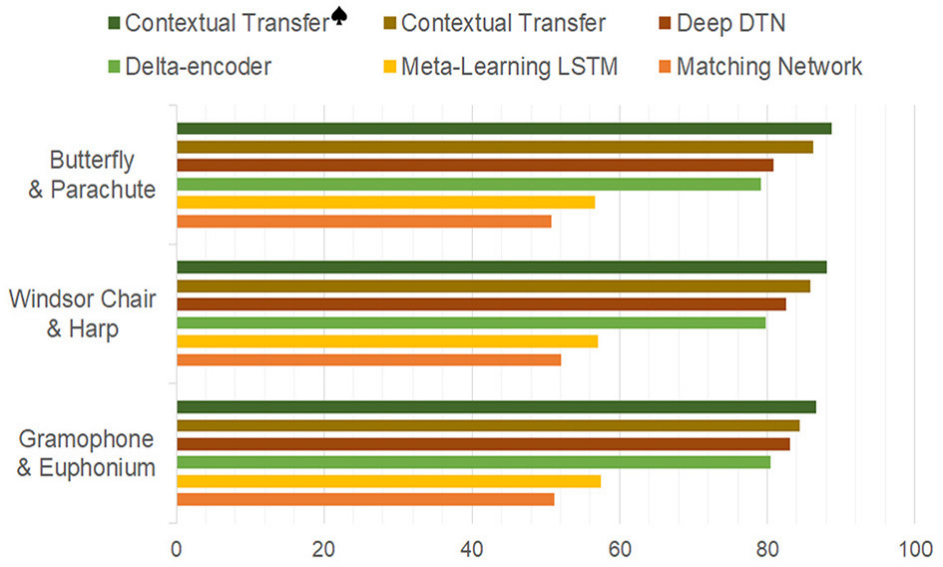
Performance comparisons with accidental similarity added.

Therefore, extra experiments are designed so that pairs of similar categories are introduced to replace some categories in the Caltech-101 training set to test the classification accuracy of the corresponding categories, respectively. The results are illustrated in Figure 5 to show that the proposed method is adept in handling these confusions of similar samples under the help of contextual information. The figure shows the maximum accuracy under the 5 Way 5 Shot setting, and the highest accuracies that the proposed contextual transfer achieves (84.4, 85.9, and 86.3) are higher than 84.2 in the original Caltech-101, while the dataset with manually-added textual modal also achieves higher accuracy, which can further verify the introduction of contextual information to improve the accuracy of few-shot classification by suppressing the influence of sample specificity.

### 4.3. Sensitivity Analysis

Since the idea of DTL is introduced to optimize feature extractors in training the model, the effect of transfer learning on model performance should be compared by ablation experiments. Besides, the Pearson correlation coefficient is used in reformulating the similar metric in this paper. To compare whether the relaxed constraint using Pearson's correlation coefficient is superior to the ones using traditional Euclidean distance and Cosine distance, experiments are also designed in this section.

**Figure 6:**
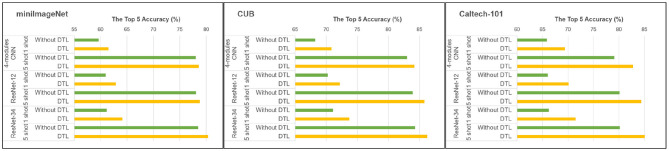
Performance comparisons using/without DTL.

As for the effect of DTL, we use the proposed scheme for training models with different architectures, including a CNN with four convolutional modules, ResNet-12, and deeper ResNet-34, on the above three datasets. The results are shown in Figure 6, which verifies the positive effect of introducing DTL. It shows that with the increase of model depth, the effect of DTL becomes more obvious. Although models with more complex structures can gain stronger feature extraction capability through more convolutional layers to improve performance, the reuse of powerful extractors and memorized convolutional layers are also able to play more important roles since there are more parameters to train under limited samples.

**Figure 7:**
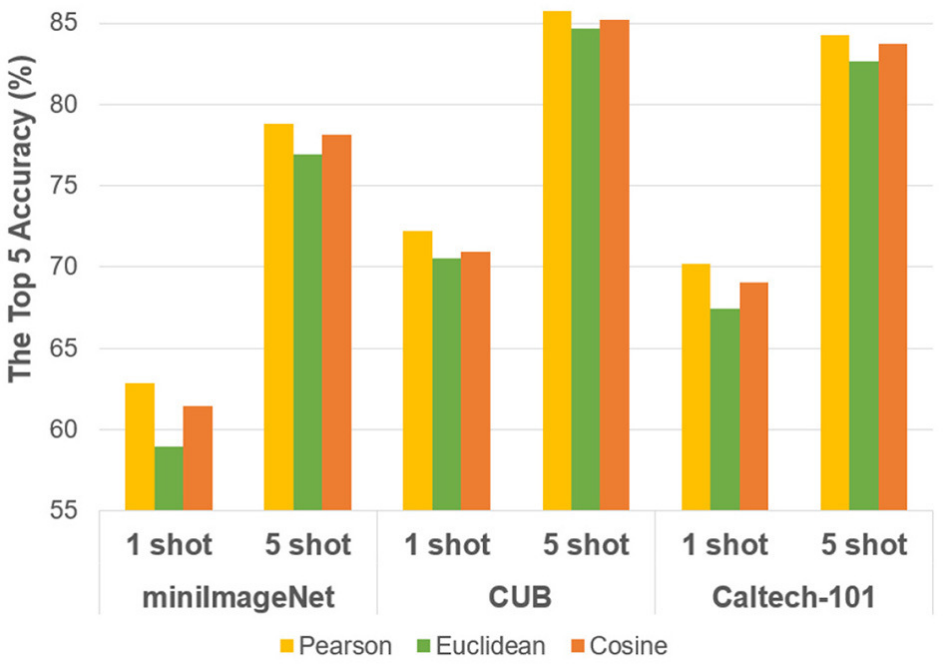
Performance comparisons with different similar metrics.

And regarding the choice of similar metrics, Figure 7 gives an illustration. The network structure of ResNet-12 is chosen for comparison, and the Pearson similarity shows better performance on either of the three datasets in 1 shot and 5 shot tasks. As discussed above in section 3, it proves that compared with Cosine and Euclidean metrics, the proposed Pearson correlation coefficient-based metric is more suitable for high-dimension and missing-dimension scenarios, since the introduction of contextual information in few-shot tasks are more likely to bring high-dimension characteristics. Meanwhile, the problem of loosening its constraints might lead to more susceptible changes in sample specificity which can also be answered by the designed contextual strategy.

## 5. Conclusion

Contextual information is a kind of effective auxiliary knowledge, which can provide valuable information for the image classification task under few-shot learning scenarios with insufficient sample diversity, to reduce the negative effect of sample specificity in extracting universal features of categories. In this paper, the ideology of deep transfer learning is introduced in that a method is proposed to realize context awareness transfer in few-shot image classification scenes, and it is also appropriate for heterogeneous data so that intra-modal and cross-modal information can be used effectively.

Concretely, first, the similarity measure in the image classification task is reformulated based on the Pearson correlation coefficient. On this basis, the reformulated similarity metric is further designed to integrate the semantic similarity in heterogeneous samples. The important semantic information extracted from textual modal and visual modal, as well as visual feature information in the background are taken into account together as a supplement, to inhibit the negative effect of specificity. Then, the DTL scheme is used to optimize the extracting process in the model for better recognition of local visual features and reorganizing the feature recognition pattern in the convolutional layers. Pre-trained powerful extractors and convolutional layers from existing models are transferred to achieve the reuse of knowledge. Finally, a series of simulation experiments are designed on the widely used and newly constructed datasets, which validate that the proposed method can effectively suppress the deviation of defining category features under a few samples, thus improving the accuracy of few-shot image classification tasks.

We believe that the proposed strategy can be utilized in various problems challenged by the scarcity of local visual information in few-shot computer vision tasks. In the future, more interesting research directions for making better use of contextual information will be explored.

## Data Availability Statement

Publicly available datasets were analyzed in this study. This data can be found here: The miniImageNet, CUB and Caltech-101 datasets using in our research are all available under open source machine learning frameworks such as PyTorch and Tensorflow for free.

## Author Contributions

ZC: provide new ideas and research concept and design. XZ: research concept and design, writing the article, and data analysis and interpretation. WH: data and opinion support and data analysis and interpretation. JG: data analysis and interpretation and research concept and design. SZ: data analysis and interpretation and critical revision of the article. All authors contributed to the article and approved the submitted version.

## Conflict of Interest

The authors declare that the research was conducted in the absence of any commercial or financial relationships that could be construed as a potential conflict of interest.
